# Short-Term Proton Pump Inhibitor Use and Hepatic Encephalopathy Risk in Patients with Decompensated Cirrhosis

**DOI:** 10.3390/jcm8081108

**Published:** 2019-07-25

**Authors:** Yi-Chun Kuan, Kuang-Wei Huang, Cheng-Li Lin, Jiing-Chyuan Luo, Chia-Hung Kao

**Affiliations:** 1 Department of Neurology, Taipei Neuroscience Institute, Taipei Medical University, Taipei 11031, Taiwan; 2 Institute of Epidemiology and Preventive Medicine, College of Public Health, National Taiwan University, Taipei 10617, Taiwan; 3 Center for Evidence-Based Health Care, Taipei Medical University-Shuang Ho Hospital, New Taipei City 23561, Taiwan; 4 Department of Neurology, Shuang Ho Hospital, Taipei Medical University, New Taipei City 23561, Taiwan; 5 Department of Neurology, School of Medicine, College of Medicine, Taipei Medical University, Taipei 11031, Taiwan; 6 Division of Gastroenterology, Department of Internal Medicine, Taipei Beitou Health Management Hospital, Taipei 11252, Taiwan; 7 Management Office for Health Data, China Medical University Hospital, Taichung 40402, Taiwan; 8 College of Medicine, China Medical University, Taichung 40402, Taiwan; 9 Division of Gastroenterology, Department of Internal Medicine, Taipei Veterans General Hospital, Taipei 11217, Taiwan; 10 Graduate Institute of Biomedical Sciences and School of Medicine, College of Medicine, China Medical University, Taichung 40402, Taiwan; 11 Department of Nuclear Medicine and PET Center, and Center of Augmented Intelligence in Healthcare, China Medical University Hospital, Taichung 40402, Taiwan; 12 Department of Bioinformatics and Medical Engineering, Asia University, Taichung 41354, Taiwan

**Keywords:** proton pump inhibitor, hepatic encephalopathy, Short-term Registry for Catastrophic Illness Patient Database

## Abstract

Objective: A window period of approximately 3–6 months is usually adopted in studies that evaluate hepatic encephalopathy (HE) risk in proton pump inhibitor (PPI) users. However, HE risk after short-term PPI exposure remains unclear. We explored the effect of short-term PPI exposure using a case-crossover study design. Design: Records of patients with decompensated cirrhosis who had received an HE diagnosis were retrieved from the National Health Insurance Research Database. PPI use rates were compared for case and control with window periods of 7, 14, and 28 days. The adjusted self-matched odds ratio (OR) and 95% confidence interval (CI) from a conditional logistic regression model were used to determine the association between PPI use and HE risk. Results: Overall, 13 195 patients were analyzed. The adjusted OR for HE risk after PPI exposure was 3.13 (95% CI = 2.33–4.20) for the 7-day window, 4.77 (95% CI = 3.81–5.98) for the 14-day window, and 5.60 (95% CI = 4.63–6.78) for the 28-day window. All PPI categories, except omeprazole and pantoprazole, were associated with an increased HE risk. Irrespective of other precipitating factors, such as recent gastrointestinal bleeding or infection, PPI significantly increased HE risk. Conclusion: Short-term PPI use is significantly associated with HE in patients with decompensated cirrhosis. Physicians should use PPI in these patients for appropriate indications, and carefully monitor signs of HE even after short-term exposure. Owing to the limitations of retrospective design in the current study, further study is warranted to confirm our findings.

## 1. Introduction

Hepatic encephalopathy (HE) is a serious neuropsychiatric complication of liver cirrhosis. The clinical spectrum ranges from minimal HE to overt HE, which may result in altered consciousness or even coma [1]. HE development is associated with short survival in patients with cirrhosis [2,3]. Additional factors are involved in triggering HE, and identifying these factors early is essential to treat HE effectively. These predisposing factors include electrolyte imbalance, high protein intake, gastrointestinal (GI) bleeding, infection, constipation, and medication such as benzodiazepines or narcotics [4,5,6,7,8].

Proton pump inhibitors (PPIs) are the most widely used medication worldwide for managing acid-related disorders, including gastroesophageal reflux disease and peptic ulcer disease. However, PPI may predispose patients to small bowel bacterial overgrowth (SIBO) and bacterial translocation [9]. SIBO is common in liver cirrhosis, which can be correlated with its severity and linked with minimal and overt HE [10].

Several recent studies have raised concerns, but they have shown conflicting results regarding the possible association between PPI and HE in patients with cirrhosis [11,12,13,14,15,16]. PPI exposure with approximately 3- to 6-month window [12,17] or vaguely defined window [11,13,14,15] has been often used to evaluate HE risk in chronic PPI users. To date, HE risk in patients with decompensated cirrhosis after short-term PPI exposure remains uncertain. Furthermore, the existence of residual confounding factors from observational epidemiology with odds ratios (ORs) <3 should be considered before any suggestion of causality is entertained [18]. To overcome the potential confounding variables not generally available in population studies and to evaluate the effect of short-term PPI exposure, we conducted a case-crossover study in patients with decompensated cirrhosis diagnosed with HE from Taiwan’s nationwide population-based claims database.

## 2. Methods

### 2.1. Case-Crossover Design

**Figure 1:**
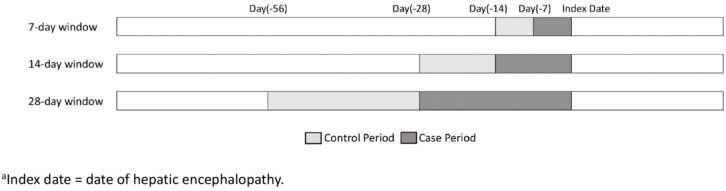
Illustration of case-crossover design to evaluate hepatic encephalopathy risk after short-term proton pump inhibitor (PPI) exposure.

The case-crossover design, first introduced by Maclure in 1991, is a research method for studying transient effects on acute event risk [19]. The case-crossover study design was devised to assess the relationship between transient exposures and acute outcomes in situations where the control series of a case-control study is difficult to achieve. Each individual patient serves as their own control in the case-crossover design (Figure 1). Therefore, stable confounders that are unknown, poorly measured, or cannot be measured eliminate each other.

### 2.2. Data Source and Study Population

Launched in 1995, the National Health Insurance (NHI) program in Taiwan provides comprehensive medical care and covers >99% of the Taiwanese population (23.75 million people). The Taiwan National Health Insurance Research Database (NHIRD) is a large medical claims database and contains the data of beneficiaries enrolled in the Taiwan NHI program. It contains information regarding disease diagnosis coded according to the International Classification of Diseases, Ninth Revision, Clinical Modification (ICD-9-CM) as well as treatment procedures, service dates, and medical costs of beneficiaries and the names of nearly 20,000 prescription drugs that they have used. All data are anonymous and de-identified using encrypted and unique personal identification numbers to make the NHI reimbursement data suitable for public research.

From the NHIRD, we selected patients who had advanced decompensated cirrhosis (ICD-9-CM 571.2, 571.5, and 571.6). Decompensated cirrhosis was defined as the development of: (1) cirrhosis with refractory ascites; (2) cirrhosis with hepatorenal syndrome; or (3) cirrhosis with esophageal or gastric varices with bleeding. The diagnoses were confirmed according to patients’ inclusion in the NHIRD. Adult patients (≥18 years old) with newly diagnosed HE (ICD-9-CM 572.2) were included for analysis. Cirrhosis etiology, hepatic decompensation at enrollment, and medical comorbidities in the past 3 months, such as GI bleeding, intra-abdominal infection, pneumonia, and urinary tract infection, defined according to ICD-9-CM codes, were included for analysis. The Charlson comorbidity index was used to assess comorbidity severity.

### 2.3. Ethics Statement

The NHIRD encrypts patient personal information to protect privacy and provides researchers with anonymous identification numbers associated with relevant claims information, including sex, date of birth, medical services received, and prescriptions. Therefore, patient consent is not required to access the NHIRD. This study was approved to fulfill the condition for exemption by the Institutional Review Board (IRB) of China Medical University (CMUH-104-REC2-115-CR3). The IRB also specifically waived the consent requirement.

### 2.4. Data Processing and Statistical Analysis

Descriptive data are expressed as mean ± standard deviation (SD) for continuous variables and as numbers of cases and percentages for categorical variables. The OR of the 7-day window for HE risk after exposure to a specific drug was estimated by the ratio of patients who were exposed to that drug during the 7-day case period (1–7 days before the index date) to patients who were exposed only during the 7-day control period (8–14 days before the index date). For case-crossover analyses, conditional logistic regression models were used to estimate the OR and their 95% confidence intervals (95% CIs). In the multivariate analysis, an adjusted OR was estimated after controlling for cirrhosis etiology, hepatic decompensation at enrollment, Charlson Comorbidity Index score, and medical comorbidities in the past 3 months. We performed a sub-analysis to investigate the association between the individual PPI use and HE risk after stratifying by each PPI category (pantoprazole, lansoprazole, omeprazole, esomeprazole, and rabeprazole). Subgroup analyses were performed by stratifying characteristics such as gender, age (≤49, 50–64, and ≥65 years), and medical comorbidities in the past 3 months. All data analyses were performed using SAS version 9.4 (SAS Institute Inc., Cary, NC, USA). A two-tailed *p* value of <0.05 was considered statistically significant.

## 3. Results

Overall, data of 13,195 patients aged ≥ 18 years with new-onset HE episode were retrieved from the NHIRD. The mean age at HE onset was 56.6 years (SD = 13.2), and 70.3% of the study participants were male. Furthermore, most of the participants had hepatic decompensation at enrollment, with 70.4% patients having varices or a history of varices bleeding followed by 60.0% and 0.6% patients having ascites and hepatorenal syndrome, respectively. The baseline demographic and clinical characteristics of the included patients are summarized in Table 1.

Table 2 presents the effect of PPI use on HE risk with different window periods. Overall, PPI use was associated with increased HE risk in patients with decompensated cirrhosis after adjusting for potential confounding factors. During the 7-, 14-, and 28-day window periods, PPI users had a 3.13- (95% CI = 2.33–4.20), 4.77- (95% CI = 3.81–5.98), and 5.60-fold (95% CI = 4.63–6.78) increased HE risk, respectively. Stratified by each individual PPI, the use of lansoprazole (OR = 4.38; 95% CI = 2.76–6.97), esomeprazole (OR = 5.14; 95% CI = 2.30–11.5), and rabeprazole (OR = 3.62; 95% CI = 1.02–12.9), but not omeprazole (OR = 1.85; 95% CI = 0.86–3.98) and pantoprazole (OR = 1.36; 95% CI = 0.72–2.58), was associated with an increased HE risk (Table 3). In the subgroup analyses, HE risk did not differ with age (age ≤ 49, OR = 1.61, 95% CI = 1.26–2.05; 50 ≤ age ≤ 64, OR = 2.00, 95% CI = 1.59–2.52; age ≥ 65, OR = 2.37, 95% CI = 1.82–3.09) or gender (female, OR = 2.05, 95% CI = 1.57–2.68; male, OR = 1.91, 95% CI = 1.62–2.25). We also tested the effect of PPI use on patients with or without precipitating factors for HE, such as recent GI bleeding and infection. Overall, PPI use significantly increased HE risk in patients with and without precipitating factors, with a higher risk trend in patients with precipitating factors as compared with those without (Table 4).

## 4. Discussion

In the current population-based case-crossover study, we observed that short-term PPI exposure is associated with a significant HE risk in patients with decompensated cirrhosis in different window periods (7–28 days).

First reported in 2014, Lin et al. found that HE occurrence is associated with hyponatremia (OR = 6.318, 95% CI = 2.803–14.241) and PPI use (OR = 4.392, 95% CI = 1.604–12.031) in patients with acute-on-chronic liver failure [15]. In recent years, 2 large studies from Denmark [11] and Taiwan [12] have again raised concerns about the possible association between PPI and HE in patients with cirrhosis. However, some limitations exist, such as small patient numbers from Lin et al., potential existence of residual confounding factors due to OR < 3 from Tsai et al. [12], and increased risk of bias due to enrollment of only those patients who had ascites and were treated with satavaptan or other diuretic drugs from Dam et al. [11]. Furthermore, PPI exposure has been defined vaguely in different studies. Moreover, only Tsai et al. found a dose-dependent HE risk among PPI users with cirrhosis. In previous studies, some confounders including lifestyle differences such as alcohol drinking, underlying comorbidities, and disease severity were not fully considered or were unavailable in research databases. Lifestyle factors such as alcoholism were strongly related to HE development in patients with decompensated cirrhosis or acute-on-chronic liver failure [20]. Uneven distribution of reported comorbidities between case and control groups may adversely affect the final results [12,15]. To overcome these potential biases, we conducted a case-crossover study [19] to look at the possible influence of short periods of prior PPI use over 3 different intervals up to 28 days. With this measurement, stable confounders that are unknown, poorly measured, or cannot be measured would be eliminated. However, considering the large sample size recruited from the NHIRD, sufficient power for statistical analyses would be obtained.

We observed that PPI exposure was associated with an elevated HE risk in all 3 window periods. The risk was the highest for the 28-day window (OR = 5.60) followed by the 14-day window (OR = 4.77) and 7-day window (OR = 3.13). The trend of increasing the window period may support the finding of dose-response relationship found by Tsai et al. Some studies have indicated that age is an additional risk factor for HE [11,21]. Subgroup analyses of the current case-crossover study indicated that PPI use independently increased HE risk in different age groups with increasing trend. In current study, we found esomeprazole, rabeprazole and lansoprazole were associated with risk of HE, but not omeprazole and pantoprazole. Esomeprazole and rabeprazole were more potent on the effect of gastric acid suppression [22]. Gastric acid suppression may play a role of SIBO, which contribute to further HE. No significant association of pantoprazole and omeprazole with HE may be due to their relatively low potency for gastric acid suppression [22], and pharmacokinetic studies have shown that lansoprazole may penetrate the blood-brain barrier [23]. Furthermore, HE could be triggered by the accumulation of drugs or toxic metabolites through PPI by modulating blood–brain barrier drug transport [24]. Therefore, these may explain why some PPIs are implicated in the induction of HE in current study. GI bleeding and infection were considered crucial precipitating factors for HE, and they may affect the statistical results [6,7,8]. These factors were not measured [11] or have been unevenly distributed [12,15] in previous cohort or case-control studies. Studies have shown conflicting results regarding the association between PPI and HE after adjustment of these precipitating factors [13]. Therefore, we performed subgroup analyses through stratification of patients with or without these precipitating factors for clarifying the effect of PPI exposure. Overall, PPI use still significantly increased HE risk in patients with decompensated cirrhosis without recent GI bleeding (adjusted OR = 1.42; 95% CI = 1.29–1.56) or infection such as intra-abdominal infection (adjusted OR = 1.43; 95% CI = 1.31–1.57), pneumonia (adjusted OR = 1.46; 95% CI = 1.34–1.59), or urinary tract infection (adjusted OR = 1.45; 95% CI = 1.33–1.58). These findings, along with a recently published study from Tsai et al. [12], may be interpreted as the risk of HE in such conditions is attributed to PPI per se rather than precipitating factors. Moreover, the greater risk of HE in PPI users concomitant with precipitating factors as compared to those without may support the additive influence each other.

The mechanism underlying the association between the PPI and HE risk may be complex. In patients with cirrhosis, pathological bacterial translocation involving impaired intestinal motility, SIBO, intestinal barrier dysfunction, systemic inflammation, and altered gut flora and their by-products, such as ammonia and endotoxins, may play a key role in HE pathogenesis [10]. First, PPI may increase SIBO incidence [9], which is associated with minimal HE occurrence [25,26]. Second, PPI delays gastric motility and emptying [27], which can lead to SIBO caused by stasis of food and bacteria in the upper GI tract. Third, PPI therapy inhibits neutrophil-endothelial cell interactions [28] and impaired mucosal antimicrobial host defense mechanisms, which contribute to bacterial translocation development in cirrhosis [29]. Fourth, PPI has been linked with altered gut microbiota [30,31], which is associated with cirrhosis and its complications [32]. Finally, apart from microbiota-dependent mechanisms, PPI may cause drugs or toxic metabolites to accumulate in the brain through P-glycoprotein drug-drug interaction at the blood-brain barrier [24].

Owing to several advantages of the current study, the results have increased validity. First, a relatively large number of patients and population-based design, which involved data from the NHIRD, provided generalizable findings. Second, to increase the diagnostic accuracy for liver cirrhosis, we included patients with advanced decompensated cirrhosis who met the diagnoses definition (mentioned in the Methods section) confirmed according to the patients’ inclusion in the NHIRD. Third, the case-crossover design eliminates within-individual time-invariant variables. However, the current study has several limitations. First, this was a retrospective design study, and the study population was selected from a claims-based data set. Some selection biases may exist; therefore, the results must be extrapolated with caution. Second, we identified patients with HE on the basis of ICD-9-CM code. Overt HE probably accounts for the majority HE, and minimal HE may be missed as it lacks obvious clinical signs and is usually determined through psychometric and neurophysiological tests [4,33]. Third, the exact indications for PPI prescriptions were unknown in the current study. Furthermore, these conditions may contribute to HE development independently of PPI usage. For example, GI bleeding or stress ulcers caused by infection would potentially lead to HE. However, we performed subgroup analyses by stratifying patients with or without these precipitating conditions for clarifying the effect of PPI exposure. Fourth, though the case-crossover design could automatically control for all time-invariant confounders, it is likely that our results were still confounded by time-variant factors, such as hyponatremia and excessive protein intake, which are not available in the NHIRD.

In conclusion, short-term (7–28 days) exposure to PPI is associated with a significantly increased HE risk in patients with decompensated cirrhosis, regardless of age, gender, and recent precipitating comorbidities. Physicians should use PPI in these patients for appropriate indications and carefully monitor HE signs even after short-term exposure. Due to the limitations of retrospective design in our study, further studies, such as prospective or randomized controlled trials, may be warranted to confirm our findings.

## Figures and Tables

**Table 1:** Demographic data of patients with decompensated cirrhosis and hepatic encephalopathy. HBV: hepatitis B virus; HCV: hepatitis C virus.

Characteristic	Cases (*N* = 13,195)	
	Mean	SD
Age (years)	56.6	13.2
	*N*	%
Gender		
Male	9271	70.3
Female	3924	29.7
Cirrhosis etiology		
HBV-related	4702	35.6
HCV-related	3982	30.2
Alcohol-related	7351	55.7
Hepatic decompensation at enrollment		
Ascites	7920	60.0
Varices or varices bleeding	9284	70.4
Hepatorenal syndrome	79	0.60
Charlson Comorbidity Index score		
0	315	2.39
1	3680	27.9
2	5709	43.3
3+	3491	26.5
Medical comorbidities in recent 3 months		
GI bleeding	3151	23.9
Intra-abdominal infection	955	7.24
Pneumonia	271	2.05
Urinary tract infection	275	2.08

**Table 2:** Hepatic encephalopathy risk associated with current proton pump inhibitor use.

	Case *N* = 13,195		Control *N* = 13,195		Crude		Adjusted ^a^	
					OR	95% CI	OR	95% CI
7 day window Proton pump inhibitor	1779	13.5%	977	7.40%	2.18	1.99–2.39	3.13	2.33–4.20
14 day window Proton pump inhibitor	2459	18.6%	1192	9.03%	2.82	2.59–3.08	4.77	3.81–5.98
28 day window Proton pump inhibitor	3155	23.9%	1268	9.61%	3.86	3.55–4.21	5.60	4.63–6.78

^a^ Adjusted for cirrhosis etiology, hepatic decompensation at enrollment, Charlson Comorbidity Index score, and medical comorbidities in recent 3 months

**Table 3:** Hepatic encephalopathy risk associated with different proton pump inhibitors for a 14-day window period.

	Case *N* = 13,195		Control *N* = 13,195		Crude		Adjusted ^a^	
					OR	95% CI	OR	95% CI
Pantoprazole	299	2.27%	178	1.35%	1.92	1.55–2.37	1.36	0.72–2.58
Lansoprazole	722	5.47%	397	3.01%	2.16	1.87–2.48	4.38	2.76–6.97
Omeprazole	255	1.93%	140	1.06%	2.29	1.79–2.94	1.85	0.86–3.98
Esomeprazole	374	2.83%	195	1.48%	2.30	1.88–2.81	5.14	2.30–11.5
Rabeprazole	129	0.98%	67	0.51%	2.55	1.77–3.68	3.62	1.02–12.9

^a^ Adjusted for cirrhosis etiology, hepatic decompensation at enrollment, Charlson Comorbidity Index score, and medical comorbidities in recent 3 months.

**Table 4:** Hepatic encephalopathy risk associated with proton pump inhibitor for a 28-day window period stratified by current medical comorbidities.

	Case *N* = 13,195	Control *N* = 13,195	Crude		Adjusted ^a^	
			OR	95% CI	OR	95% CI
GI bleeding						
Yes	605	247	2.91	2.35–3.61	2.71	2.10–3.49
No	2550	1021	2.97	2.75–3.21	1.42	1.29–1.56
Intra-abdominal infection						
Yes	183	60	3.86	2.52–5.90	3.93	2.32–6.65
No	2972	1208	2.92	2.72–3.14	1.43	1.31–1.57
Pneumonia						
Yes	50	20	1.96	0.96–3.99	1.63	0.69–3.85
No	3105	1248	2.96	2.76–3.18	1.46	1.34–1.59
Urinary tract infection						
Yes	26	8	4.62	1.72–12.4	10.8	2.58–45.6
No	3129	1260	2.95	2.75–3.17	1.45	1.33–1.58

^a^ Mutually adjusted for cirrhosis etiology, hepatic decompensation at enrollment, and Charlson Comorbidity Index score.
